# Effects of Childhood Stressors on Memory: Mediating Role of Psychological Resilience

**DOI:** 10.1093/geroni/igaf122.3230

**Published:** 2025-12-31

**Authors:** Guoping Jin

**Affiliations:** University of Pittsburgh, Pittsburgh, Pennsylvania, United States

## Abstract

Memory decline in later life is a growing public concern. While prior research suggests that childhood stressors are associated with lower cognitive functioning in older adults, less is known about their specific impact on memory performance and the potential mechanisms underlying this relationship. Drawing on the theoretical framework of resilience, this study investigated the long-term effects of childhood stressors on memory trajectories in later life, with a particular focus on the mediating role of psychological resilience. Data were from adults aged 65 and over who completed the Leave Behind Questionnaire in the 2006/2008 wave of the Health and Retirement Study (n = 9,069). Path analysis and latent growth curve modeling within a structural equation modeling framework were used to examine the direct and indirect pathways linking childhood stressors to memory trajectories through psychological resilience. The results indicated that respondents who reported a greater number of childhood stressors had higher baseline memory performance (β = 0.12, p < .001). However, childhood stressors were not significantly associated with memory decline over time. Moreover, psychological resilience partially mediated the relationship between childhood stressors and baseline memory (indirect effect = -0.01, p < .05). These findings highlighted the complex role of childhood stressors in shaping later-life memory function, suggesting that early-life adversity may foster cognitive adaptations while also undermining psychological resilience. Future research should explore additional mediators and potential moderators to better understand the mechanisms linking childhood experiences to memory function. Other implications for practice and interventions are discussed.

